# Sudden, Transient Intraoperative Hypotension During the Use of the Cantilever Technique for Correction of Adult Spine Deformity

**DOI:** 10.7759/cureus.13835

**Published:** 2021-03-11

**Authors:** Kentaro Mataki, Masao Koda, Toru Funayama, Hiroshi Takahashi, Masashi Yamazaki

**Affiliations:** 1 Department of Orthopaedic Surgery, University of Tsukuba, Faculty of Medicine, Tsukuba, JPN

**Keywords:** transient sudden hypotension, posterior spinal correction, anterior longitudinal ligament rupture, decrease of venous return, inferior vena cava compression and stretching

## Abstract

Intraoperative hypotension is a common but critical complication of spinal surgery. However, it is uncommon to experience sudden transient intraoperative hypotension in patients undergoing surgery for adult spine deformity (ASD) without the presence of major vascular injury, spinal cord injury, or cardiac events. We report a patient who experienced sudden transient intraoperative hypotension during the use of the cantilever technique for correction of an ASD. A 58-year-old woman underwent two-stage surgery (anterior correction followed by posterior fusion) for an ASD that caused low back pain. During the posterior fusion procedure, she experienced sudden transient intraoperative hypotension during the use of a cantilever technique. As soon as we paused the use of this technique, her hypotension resolved. Postoperative radiography revealed excessive segmental lordosis at the L4/5 level, suggesting an accidental rupture of the anterior longitudinal ligament (ALL). We believe that the mechanism of our patient’s sudden hypotension was a decrease in venous return due to compression and stretching of the inferior vena cava at the time of rod application when the use of the cantilever technique caused ALL rupture. Sudden hypotension during posterior spinal correction surgery is possible, especially in patients with a ruptured ALL.

## Introduction

Over the last several decades, significant advances have been made in assessing and managing patients with spinal deformities. The primary goal of surgery for the adult spinal deformity (ASD) is to restore sagittal balance, which is closely associated with pain and disability [1, 2]. Lateral lumbar interbody fusion (LLIF) is widely used for ASD [3-5], and it is effective for the correction of moderate sagittal plane deformities when used in combination with traditional open posterior techniques that employ facet osteotomies and rod cantilever techniques [6]. However, the rupture of the anterior longitudinal ligament (ALL) can occur accidentally during LLIF and posterior correction and can greatly affect sagittal correction [7]. There are a few reports of fatal complications associated with LLIF; these involve vascular injury due to ALL rupture [8]. However, there are no reports describing intraoperative hypotension during posterior spine correction surgery with accidental ALL rupture. We report a patient with sudden transient intraoperative hypotension coinciding with the use of the cantilever technique during posterior spinal correction surgery that involved accidental rupture of the ALL.

## Case presentation

**Figure 1:**
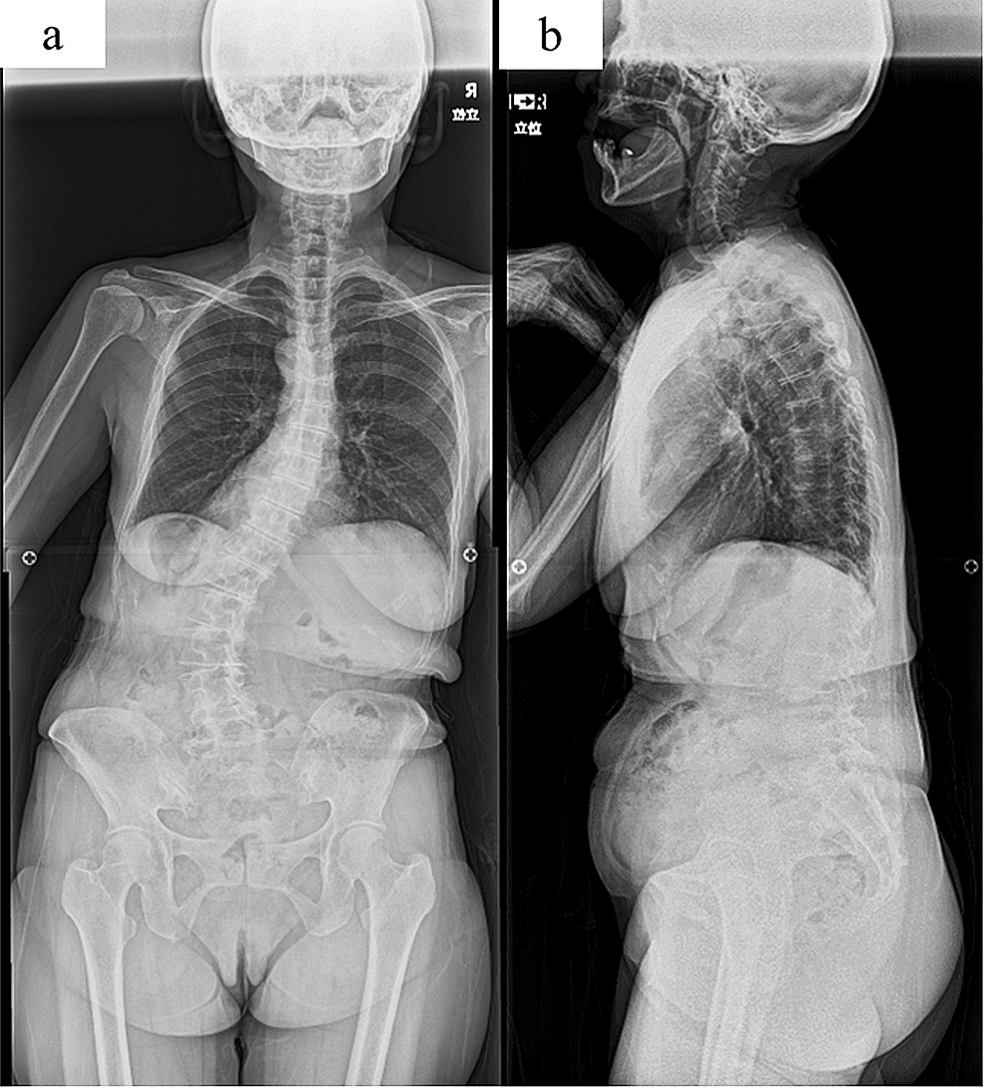
Preoperative images Preoperative posterolateral (a) and lateral (b) standing radiographs showing degenerative lumbar scoliosis and kyphotic deformities with poor global alignment.

A 58-year-old woman presented to our hospital complaining of several years of progressive back pain that occurred with standing and walking. She had no significant medical history and was taking no medications. She did not have scoliosis during adolescence. Standing radiography showed scoliotic and kyphotic deformities (central sacral vertical line, 55 mm; pelvic incidence, 65°; lumbar lordosis, 25°; sagittal vertical axis, 90 mm) with poor global alignment (Figure 1). She had no vascular abnormality or vascular calcification in the preoperative examination. 

We planned a two-stage procedure, consisting of an anterior correction followed by posterior fusion. The first stage consisted of LLIF using the extreme lateral interbody fusion (XLIF) technique, using a right-sided approach, between L3/4 and L4/5. There were no complications, and her lumbar lordosis was corrected to 35°.

**Figure 2:**
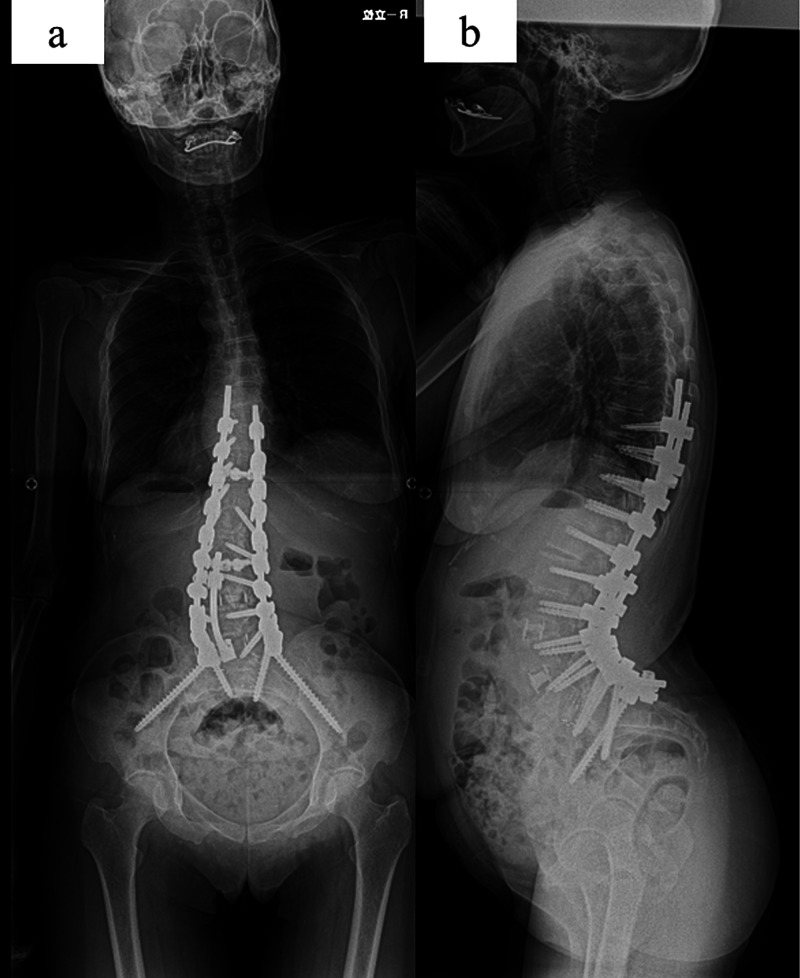
Postoperative images Postoperative posterolateral (a) and lateral (b) standing radiographs. The patient underwent combined anterior (L3/4, L4/5) extreme lateral interbody fusion and posterior fixation (T9-ilium), restoring significant lumbar lordosis and improving global coronal and sagittal balance.

Two weeks later, we performed posterior corrective fusion (T9 to ilium) using a traditional open posterior technique with a grade 2 osteotomy [9], both inferior and superior facets of articulation at a given spinal segment were resected, at L1/2, L3/4, L4/5, and L5/S (Figure 2).

**Figure 3:**
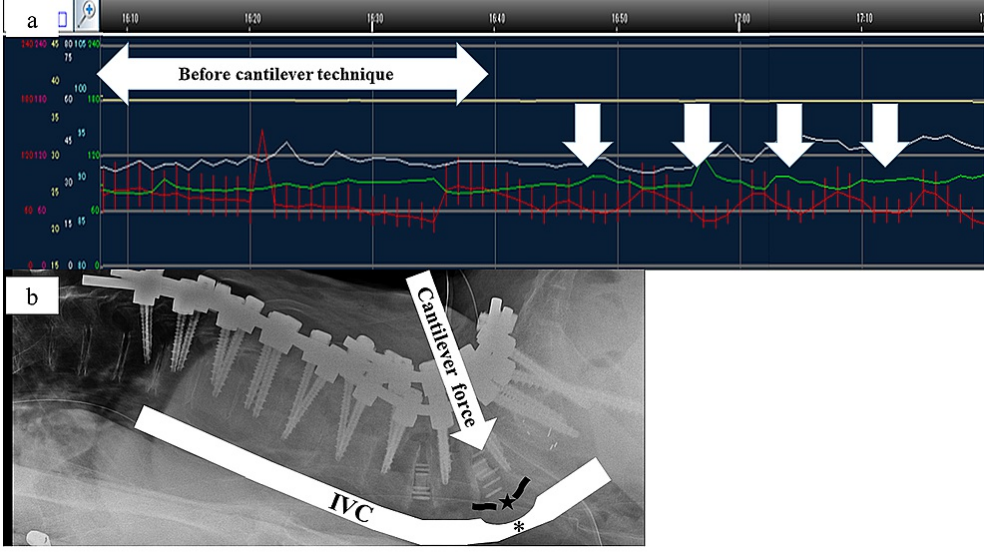
Intraoperative monitoring of mean arterial pressure The red waveform indicates continuous intraoperative monitoring of mean arterial pressure. Before the cantilever technique, the mean arterial pressure was stable. The white arrows show the timing of the cantilever technique (a). Compression and stretching of the inferior vena cava (*) occurs with the use of the cantilever technique (white arrow) and with rupture of the anterior longitudinal ligament at L4/5 (★) (b). IVC - inferior vena cava

We also employed a rod cantilever technique. While using the cantilever technique to fix the lower lumbar spine, the patient experienced transient hypotension without tachycardia, with a mean arterial pressure of 50 mm Hg. We paused the cantilever technique, and the hypotension resolved. However, it recurred when the cantilever technique was resumed (Figure 3).

There was no active bleeding at the surgical site and no change in electrocardiographic monitoring. In other words, there was no evidence of vascular injury or cardiac event. We continued carefully with rod fixation, creating a slightly decreased degree of lumbar lordosis than at the initial rod application, and the hypotension did not recur.

After this second surgery, the patient was in stable condition without hypotension or abdominal pain. Laboratory analysis revealed that her pre-existing mild anemia was not progressing (hemoglobin 12.1 g/dL before posterior fusion; 11.7 g/dL after fusion).

**Figure 4:**
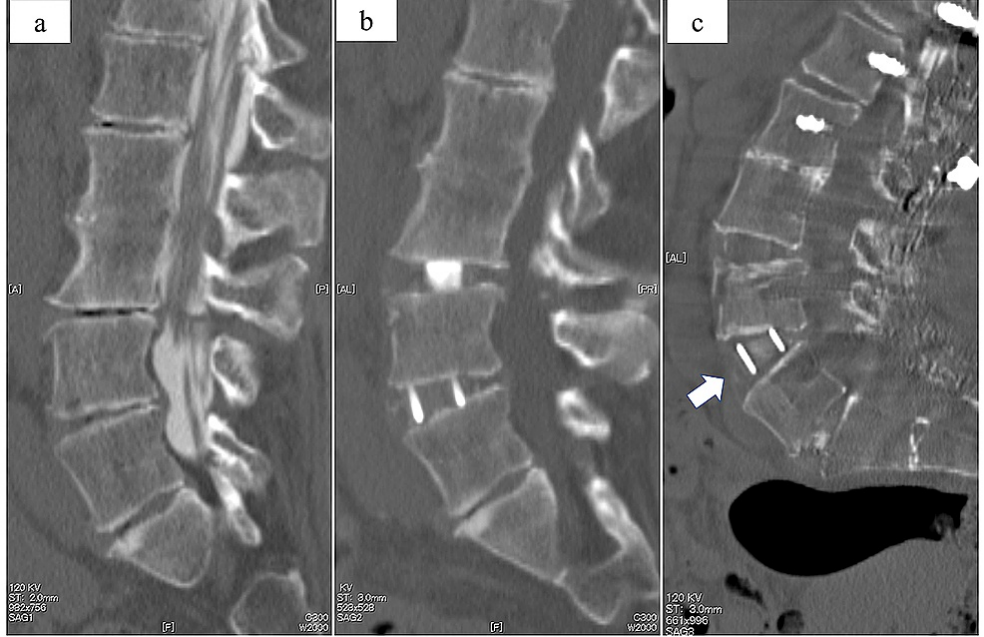
The segmental lordosis at L4/5 Computed tomography of the lumbar spine, sagittal plane preoperative imaging. The segmental lordotic angle at L4/5 was 9.7° preoperatively (a). After anterior fixation (XLIF) (b), the angle was 14°. After posterior fixation (c), the angle was 40° postoperatively with rupture of the anterior longitudinal ligament (white arrow).

The patient’s postoperative lumbar lordosis was corrected to 70°. Notably, there was 40° of segmental lordosis at L4/5, suggesting accidental ALL rupture (Figure 4).

## Discussion

In surgery for spinal deformities, the strong force used during LLIF and posterior corrective fusion is applied to the spine and surrounding structures, such as the ALL, blood vessels anterior to the vertebrae, and celiac organs. Nakashima et al. report that ALL rupture occurs accidentally during 6.5% of LLIF surgeries that involve posterior correction. The acquired segmental lordotic angles achieved by posterior fixation at levels with ALL rupture are reportedly significantly larger than at those at levels without ALL rupture [7]. In our patient, the acquired segmental lordotic angle at L4/5 changed from 9.7° to 40°. We, therefore, suspected accidental ALL rupture during LLIF or posterior correction procedure.

During spinal correction surgery, sudden intraoperative hypotension may occur as a result of major vascular injury, neurophysiological dysfunction due to spinal cord injury, or cardiac events [8, 10]. However, there are no reports besides ours that describe sudden transient intraoperative hypotension during spinal correction surgery that coincides with using a rod cantilever technique. We believe that the mechanism of the sudden transient intraoperative hypotension in our patient was decreased venous return caused by compression and stretching of the inferior vena cava (IVC). As a result of the accidental ALL rupture, the IVC at the L4/5 level suffered further compression and stretching when a strong correctional force was applied during the cantilever technique. Furthermore, it may be difficult for stress to escape due to elongation and compression because the inferior vena cava at the L4/5 level is near the bifurcation, which is also a factor that caused the impaired return of the inferior vena cava. This mechanism is similar to that of the supine hypotensive syndrome (also referred to as IVC compression syndrome), which results when the gravid uterus compresses the IVC while a pregnant woman is in a supine position, leading to decreased central venous return. Women who become symptomatic should move into the left lateral position, and symptoms will resolve rapidly [11, 12]. In our patient, the intraoperative hypotension recovered rapidly as soon as the cantilever technique was paused.

Following an episode of hypotension, tachycardia usually occurs as a compensatory autonomic nervous system reflex [13]. However, our patient experienced transient hypotension without tachycardia. We think that pausing the cantilever technique within a few minutes of the onset of hypotension before the autonomic nervous system reflex could occur prevented tachycardia.

## Conclusions

When ALL rupture occurs during posterior fusion surgery to correct kyphosis using a cantilever technique, it may cause intraoperative hypotension due to IVC compression and stretching. Surgeons should consider ALL rupture when patients experience sudden transient intraoperative hypotension while using the cantilever technique, and they should pause the use of the technique.
